# Minimum Sizes of Respiratory Particles Carrying SARS-CoV-2 and the Possibility of Aerosol Generation

**DOI:** 10.3390/ijerph17196960

**Published:** 2020-09-23

**Authors:** Byung Uk Lee

**Affiliations:** Aerosol and Bioengineering Laboratory, College of Engineering, Konkuk University, 120 Neungdong-ro, Gwangjin-gu, Seoul 05029, Korea; leebu@konkuk.ac.kr

**Keywords:** COVID-19, Middle East respiratory syndrome coronavirus, bioaerosol, aerosol, aerosol transmission, droplet, severe acute respiratory syndrome coronavirus 2, virus transmission, airborne transmission, SARS-CoV-2 bioaerosol, air infection, viral infection, MERS, nosocomial infection, respiratory particle, SARS, minimum size, aerosol suspension time, contagious disease

## Abstract

This study calculates and elucidates the minimum size of respiratory particles that are potential carriers of the severe acute respiratory syndrome coronavirus 2 (SARS-CoV-2); furthermore, it evaluates the aerosol generation potential of SARS-CoV-2. The calculations are based on experimental results and theoretical models. In the case of maximum viral-loading derived from experimental data of COVID-19 patients, 8.97 × 10^−5^% of a respiratory fluid particle from a COVID-19 patient is occupied by SARS-CoV-2. Hence, the minimum size of a respiratory particle that can contain SARS-CoV-2 is calculated to be approximately 9.3 μm. The minimum size of the particles can decrease due to the evaporation of water on the particle surfaces. There are limitations to this analysis: (a) assumption that the viruses are homogeneously distributed in respiratory fluid particles and (b) considering a gene copy as a single virion in unit conversions. However, the study shows that high viral loads can decrease the minimum size of respiratory particles containing SARS-CoV-2, thereby increasing the probability of aerosol generation of the viruses. The aerosol generation theory created in this study for COVID-19 has the potential to be applied to other contagious diseases that are caused by respiratory infectious microorganisms.

## 1. Introduction

The novel severe acute respiratory syndrome corona virus 2 (SARS-CoV-2) that emerged in Wuhan City, China, has spread worldwide. As of writing, more than thirty million cases of infection have been reported [1]. More than twenty thousand people have been infected with SARS-CoV-2 in the Republic of Korea alone [1].

To decrease the spread of COVID-19, it is important to investigate the transmission routes of SARS-CoV-2. Although SARS-CoV-2 has been detected in the stool specimen of patients, fecal–oral transmission of the virus has not been confirmed [2]. Currently, SARS-CoV-2 is considered to be mainly transmitted via respiratory droplets [3]. Generally, respiratory droplets are defined as large respiratory particles that are >5–10 μm in diameter [3]. SARS-CoV-2 can be transmitted via droplets when people are in close contact (within one meter) or owing to fomite transmission in the immediate environment [3].

However, it has been reported that small particles (<5 μm), called droplet nuclei, can be generated by human activities. These particles are suspected to be another route of airborne transmission for various viruses. Few review studies have examined the aerosol transmission of viruses [4,5]. Several discussions on SARS-CoV-2 transmission routes have been conducted from various points of views, such as fluid physics, social distancing and expiratory particle generation [6,7,8]. In addition, a multiphase turbulent gas cloud was suggested for the classification of respiratory particles instead of the dichotomy of droplet and droplet nuclei [9].

In this study, an analytic discussion of experimental studies supporting the possibility of aerosol transmission of SARS-CoV-2 is presented, along with mechanistic modeling. Specific investigations were newly conducted to calculate and analyze the size of respiratory particles containing SARS-CoV-2, along with a comparison of the experimental results. Various logical reasons are presented to support the possibility of aerosol generation for SARS-CoV-2 transmission.

## 2. Respiratory Particles

Small particles that can be airborne are defined as aerosol particles [10]. Several experimental results support the view that respiratory particles are sufficiently small for airborne transmission of microorganisms inside the particles. In a study by Johnson et al. (2011), it was reported that healthy subjects (8–15 humans) generate various particles—including respiratory droplets—of three size modes during speech and voluntary coughing (1.6, 2.5 and 145 μm, and 1.6, 1.7 and 123 μm, respectively) [11]. These particles contained very large respiratory droplets with sizes exceeding 100 μm, which fell to the ground within a few seconds. However, in the experimental results, small particles of approximately 2 μm were generated simultaneously [11] and they could remain airborne for dozens of minutes.

In another study by Lindsley et al. (2012), the sizes of aerosol particles generated by patients (9 subjects) who were infected by the influenza virus were measured [12]. The size of the generated particles ranged from 0.35 to 9 μm. Among the particles generated by the influenza-infected patients, particles with a size range of 0.35–2.5 μm were of higher number concentration. Furthermore, these particles could remain airborne for dozens of minutes to several hours [10]. Therefore, the detected particles from influenza-infected patients were estimated to be mainly airborne for significant periods.

Aerosol particles from patients (10 subjects) with an upper respiratory tract infection were measured by Lee et al. (2019) under clean air conditions [13]. The size of the particles generated from these patients ranged from <0.1 μm to 10 μm. A significant number of particles <1 μm in size were generated by the coughing patients. The results of these studies, conducted on healthy humans, influenza-infected patients and patients with upper respiratory tract infections, demonstrated that a significant amount of respiratory particles that are sufficiently small to be airborne for at least several minutes are generated. Therefore, airflow can transport these particles over time.

Additionally, the characteristics of the generated respiratory particles in the aforementioned studies were found to be related to human health conditions. For instance, in studies conducted on patients suffering from influenza and upper respiratory tract infections, the number of generated aerosol particles decreased when the subjects recovered from diseases [12,13].

## 3. Coronavirus Bioaerosols

Artificially generated aerosols carrying coronaviruses have been studied with testing their stability. First, the Middle East respiratory syndrome coronavirus (MERS-CoV) was aerosolized for 10 min and its viability was measured at 40% and 70% relative humidity (RH) conditions [14]. The results revealed that MERS-CoV was stable at 40% RH. However, the virus viability was significantly lost at 70% RH. Second, SARS-CoV-2 was aerosolized for three hours and its viability was analyzed. It was found that the virus was viable even after three hours, with limited loss of viability [15].

Coronavirus genetic materials in the air have been detected in several studies. In a study by Azhar et al. (2014) in Saudi Arabia, the MERS-CoV genome was detected in an air sample from a camel barn of an infected patient [16]. In Wuhan (China) and Nebraska (US), SARS-CoV-2 nucleic acid tests conducted on air samples gave positive results at an intensive care unit of a hospital in Wuhan (China) and in a patient room of a university medical center in Nebraska (US) [17,18]. In Florida (US), SARS-CoV-2 was detected in air samples at the Student Health Care Center at the University of Florida via RT–PCR analysis [19]. In this study in Florida, the SARS-CoV-2 concentration was estimated to be 0.87 virus genomes/L air [19]. Furthermore, in a study by Chia et al. (2020) in Singapore, SARS-CoV-2 was detected in air samples from the airborne infection isolation rooms of infected patients via RT–PCR analysis and ORFlab assay [20]. In a study by Liu (2020), SARS-CoV-2 RNA was detected in air samples from hospitals and public areas, such as department stores, in Wuhan (China) [21]. The detection of coronavirus genes in air samples implies that it is highly probable that coronavirus bioaerosols were present at the sampling locations.

In a study by Lednicky et al. (2020), the isolation of viable SARS-CoV-2 from air samples of the surroundings (2 to 4.8 m away) of patients in a hospital was reported in Florida (US) [22].

## 4. Calculation of Sizes of Respiratory Particles Containing SARS-CoV-2

The size of SARS-CoV-2 ranges from 0.07 μm to 0.09 μm [23,24]. The following calculations of the minimum sizes of particles containing SARS-CoV-2 are based on the assumption of a homogeneous distribution of viruses inside respiratory fluid particles, with a maximum size of 0.09 μm (sphere, d_virus_) for SARS-CoV-2. In addition, a spherical volume ratio was used.

$$\text{Volume ratio of viruses in released respiratory fluid}=\frac{\frac{\pi d_{virus}{}^{3}}{6}}{\frac{\pi d_{particle}{}^{3}}{6}}$$

The following calculations of aerosol suspension times are based on a falling height of one meter with a balance between gravity and Stokes’ law to determine the air friction on the aerosol particles [10].

$$\text{Aerosol suspension time}=\frac{18\;\mu }{d_{aerosol}{}^{2}\rho_{p}g}$$

Here, μ is the air viscosity, $\rho_{p}$ is the density of the aerosol particles (assumed equal to the density of water) and *g* is the gravity acceleration; *d_virus_*, *d_particle_* and *d_aerosol_* are the diameters of viruses, respiratory particles and aerosols, respectively [10].

Assuming that a respiratory particle can be completely (100%) constituted of SARS-CoV-2, the theoretical minimum size of particles containing SARS-CoV-2 is calculated to be 0.09 μm corresponding to the size of a single virion.

**Figure 1:**
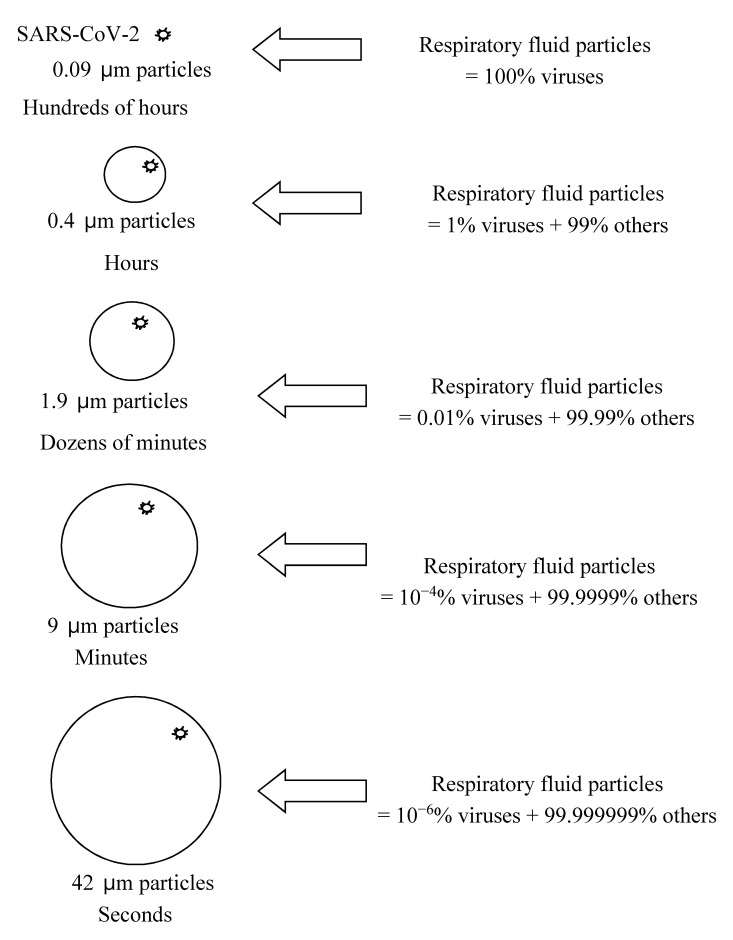
Estimated minimum size of particles (assuming homogenous distribution of viruses in released respiratory fluid particles and virus size of 0.09 μm) potentially carrying SARS-CoV-2 and corresponding aerosol suspension times.

**Figure 2:**
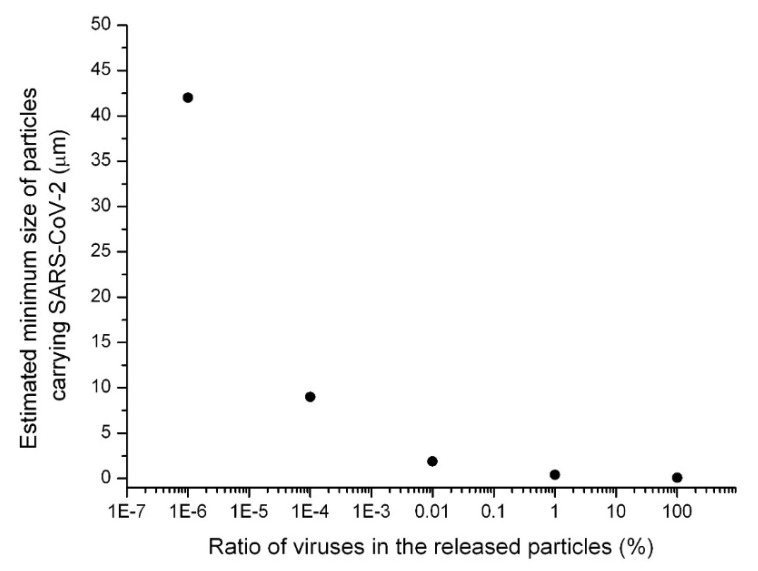
Estimated minimum size of particles (assuming homogenous distribution of viruses in released respiratory fluid particles and virus size of 0.09 μm) potentially carrying SARS-CoV-2.

If 1% of the respiratory fluid particle is occupied by SARS-CoV-2, the minimum size of the respiratory particle that can contain SARS-CoV-2 is approximately 0.4 μm. Figure 1 and Figure 2 show the calculated particle sizes via a schematic diagram and graph, respectively. If 0.01% of the respiratory fluid particle is occupied by SARS-CoV-2, the minimum size of the respiratory particle that can contain SARS-CoV-2 is approximately 1.9 μm. If only 10^−4^% of the respiratory fluid particle is occupied by SARS-CoV-2, the minimum size of the respiratory particle that can contain SARS-CoV-2 is approximately 9 μm. Furthermore, if only 10^−6^% of the respiratory fluid particle is occupied by SARS-CoV-2, the minimum size of the respiratory particle that can contain SARS-CoV-2 is approximately 42 μm. Previous studies have demonstrated that these minimum sizes could, in practice, be the sizes of actual respiratory particles [11,12,13]. In Figure 1 and Figure 2, the ratio of viruses in the released respiratory fluid particles to the total particle volume is assumed to range from 100% to 10^−6^%.

In a study by Wölfel et al. (2020), it was reported that the average ratio of viruses in the oral fluid of COVID-19 patients was 7.00 × 10^6^ copies per mL and the maximum ratio was 2.35 × 10^9^ copies per mL [25]. These experimental results can be converted to demonstrate that, on average, 2.67 × 10^−7^% of a respiratory fluid particle of COVID-19 patients is occupied by SARS-CoV-2 and then the minimum size of a respiratory particle that can contain SARS-CoV-2 is approximately 65 μm. In addition, a maximum of 8.97 × 10^−5^% of a respiratory fluid particle of COVID-19 patients is occupied by SARS-CoV-2 and then the minimum size of a respiratory particle that can contain SARS-CoV-2 is approximately 9.3 μm. The expected suspension times for these two cases were several seconds and several minutes for the average and maximum cases, respectively, under the conditions of no water evaporation on the corresponding particle surfaces. Here, the conversion from the unit of “copy per mL” based on the ratio of viruses in the oral fluid to the unit of “volume ratio” was conducted by assuming that: (1) the size of virus was 0.09 μm and (2) one copy corresponded to one virion (sphere).

This calculation of minimum particle sizes containing SARS-CoV-2 can be compared with previous experimental measurements. Studies have been conducted on the detection of SARS-CoV-2 in size-fractioned aerosol samples. In a study by Chia et al. (2020), SARS-CoV-2 genes were detected in sampled aerosol particles with diameters >4 μm and 1–4 μm [20]. In a study by Liu et al. (2020), SARS-CoV-2 was detected in submicrometer aerosol particles ranging between <0.25 and 1 μm in diameter via a droplet-digital-PCR-based detection method [21]. In a study by Liu et al. (2020), the maximum SARS-CoV-2 concentrations of 40 and 9 copies per m^3^ of air were measured in aerosol samples with diameters of 0.25–0.5 μm and 0.5–1.0 μm, respectively [21].

Based on these experimental studies obtained via size-fractioned aerosol measurements (Table 1), it can be estimated that the ratio of viruses in the oral fluid of some COVID-19 patients may be higher than the data obtained by Wölfel et al. (2020). Alternatively, it can be assumed that the respiratory particles tend to shrink after being released from humans owing to the evaporation of water on the particle surfaces, thereby making the particles smaller [10,20,21,26,27].

In a study by Wang et al. (2016), the total evaporation time of water droplets that were 25 μm in diameter ranged from 6.3 to 7.2 s (temperature = 30 °C; initial velocity of droplets = 0.3 m/s; relative humidity of ambient air = 20% to 80%) [26]. The total evaporation time of water droplets that were 10 μm in diameter was 2.8 s (temperature = 30 °C; initial velocity of droplets = 0.3 m/s; relative humidity of ambient air = 50%) [26].

If the respiratory particles are larger than 50 μm, they can only remain airborne for a few seconds; therefore, the possibility of aerosol transmission is limited. However, if the respiratory particles are smaller than 9 μm (10^−4^% condition, with a probability of one in a million), they can remain airborne for more than minutes or even hours. In such cases, aerosol transmission of viable SARS-CoV-2 could be possible. In addition, if the water evaporation on surfaces of respiratory particles is considered to occur at <80–90% relative humidity conditions, the respiratory particles that are smaller than 9 μm in diameter will be transformed into nuclei that are 0.09 μm in diameter during their airborne transmission process [26,27], and therefore, the SARS-CoV-2 bioaerosols can remain airborne for a longer time.

There are several limitations in this analysis. Viruses can be distributed nonhomogeneously inside the respiratory fluid particles; therefore, the minimum size can vary owing to the clustering of viruses in fluid particles. In addition, the genes detected in size-fractioned aerosol samples cannot be confirmed to represent viable viruses, virions. Other factors, such as air pollutants, density of virions and chemical components in respiratory fluid particles, were not considered in this analysis. For example, if the density of the virions is lower than the density of water, it will increase the aerosol suspension times of respiratory particles. Although there are limitations in the current analysis, this calculation shows that higher loads of viral shedding in respiratory fluid particles can induce the generation of smaller aerosol particles containing SARS-CoV-2; therefore, SARS-CoV-2 bioaerosol generation can occur.

## 5. Conclusions

There have been several clusters of SARS-CoV-2 infections in the Republic of Korea [28]. More than 100 people were infected by SARS-CoV-2 at a single hospital [28]. At a single telephone call service center inside a building, more than 90 employees were infected by SARS-CoV-2 [28,29]. Additionally, over 40 people were infected by SARS-CoV-2 in a single church [28]. Globally, other clusters have been reported. For example, it was reported that, in the United States, dozens of members of a choir were infected by SARS-CoV-2 after 2.5 h of rehearsal [30]. In China, clusters of infections were reported in a restaurant and a bus [31,32].

Overall, (1) the cases in which dozens of people were infected by the virus in closed environments, (2) the calculated size of the respiratory particles containing SARS-CoV-2, (3) the viability of SARS-CoV-2 bioaerosols, and (4) the detection results of SARS-CoV-2 genes in aerosol samples, all indicate that the generation of aerosols of SARS-CoV-2 is highly possible in confined environments, and that SARS-CoV-2 bioaerosols can be considered to play an important role in the pandemic occurring in 2020. Additionally, in a study by He et al. (2020), it was estimated that viral respiratory shedding in COVID-19 patients peaked on or before symptom onset, and it was reported that the measured viral load gradually decreased from the onset point of symptoms by studying the SARS-CoV-2 shedding dynamics [33]. Based on the study by He et al. (2020) and the current analysis of particle sizes containing SARS-CoV-2, it can be assumed that the generation of SARS-CoV-2 bioaerosol particles is more probable at the point of onset of symptoms in COVID-19 patients because high viral loads can increase the probability of the generation of smaller respiratory particles that can potentially carry the viruses (Figure 1 and Figure 2). The aerosol generation theory proposed in this study for COVID-19 has the potential to be applied to other contagious diseases caused by respiratory infectious microorganisms.

## Figures and Tables

**Table 1:** Minimum size of particles potentially carrying SARS-CoV-2.

Aerosol Generation	Volume Ratio of Viruses in Released Respiratory Particles	Particle Size
Lee’s theory (homogeneity assumption, without considering the decrease in sizes due to water evaporation on surfaces)	100%	0.09 μm
	1%	0.4 μm
	0.01%	1.9 μm
	10^−4^%	9 μm
	10^−6^%	42 μm
Lee’s calculations based on data in Wölfel et al. (2020) [25]	7.00 × 10^6^ copies per mL (average)	65 μm
	2.35 × 10^9^ copies per mL (maximum)	9.3 μm
Chia et al. (2020): SARS-CoV-2 genes detected in aerosols [20]		1–4 μm
Liu et al. (2020): SARS-CoV-2 genes detected in aerosols [21]		<0.25–0.5 μm
