# Correction: Lee, B.U. Minimum Sizes of Respiratory Particles Carrying SARS-CoV-2 and the Possibility of Aerosol Generation. *Int. J. Environ. Res. Public Health* 2020, *17*, 6960

**DOI:** 10.3390/ijerph182211738

**Published:** 2021-11-09

**Authors:** Byung Uk Lee

**Affiliations:** Aerosol and Bioengineering Laboratory, College of Engineering, Konkuk University, 120 Neungdong-ro, Gwangjin-gu, Seoul 05029, Korea; leebu@konkuk.ac.kr

The author would like to update a few calculation results in the Abstract and Section 4 “Calculation of Sizes of Respiratory Particles Containing SARS-CoV-2” in the previous publication [1].

**Text Correction**

It is necessary to update calculation results regarding unit conversions from copies per mL to volume ratio (%) in the original article. Corrections have been made to “Abstract” and “4. Calculation of Sizes of Respiratory Particles Containing SARS-CoV-2”:

Abstract

In the case of maximum viral-loading derived from experimental data of COVID-19 patients, 8.97 × 10^−5^% of a respiratory fluid particle from a COVID-19 patient is occupied by SARS-CoV-2. Hence, the minimum size of a respiratory particle that can contain SARS-CoV-2 is calculated to be approximately 9.3 µm.

4. Calculation of Sizes of Respiratory Particles Containing SARS-CoV-2

In a study by Wölfel et al. (2020), it was reported that the average ratio of viruses in the oral fluid of COVID-19 patients was 7.00 × 10^6^ copies per mL and the maximum ratio was 2.35 × 10^9^ copies per mL [25]. These experimental results can be converted to demonstrate that, on average, 2.67 × 10^−7^% of a respiratory fluid particle of COVID-19 patients is occupied by SARS-CoV-2 and then the minimum size of a respiratory particle that can contain SARS-CoV-2 is approximately 65 µm. In addition, a maximum of 8.97 × 10^−5^% of a respiratory fluid particle of COVID-19 patients is occupied by SARS-CoV-2 and then the minimum size of a respiratory particle that can contain SARS-CoV-2 is approximately 9.3 µm. The expected suspension times for these two cases were several seconds and several minutes for the average and maximum cases, respectively, under the conditions of no water evaporation on the corresponding particle surfaces.

**Table Correction**

It is necessary to update the calculation results of unit conversions from copies per mL to volume ratio (%) in Table 1. The corrected Table 1 appears below.

The author states that the scientific conclusions are unaffected. The original article has been updated. Related calculation details regarding unit conversions from copies per mL to volume ratio (%) were discussed extensively in the new publication [2].

## Figures and Tables

**Table 1:** Minimum size of particles potentially carrying SARS-CoV-2.

Aerosol Generation	Volume Ratio of Viruses in Released Respiratory Particles	Particle Size
Lee’s theory (homogeneity assumption, without considering the decrease in sizes due to water evaporation on surfaces)	100%	0.09 μm
	1%	0.4 μm
	0.01%	1.9 μm
	10^−4^%	9 μm
	10^−6^%	42 μm
Lee’s calculations based on data in Wölfel et al. (2020) [25]	7.00 × 10^6^ copies per mL (average)	65 μm
	2.35 × 10^9^ copies per mL (maximum)	9.3 μm
Chia et al. (2020): SARS-CoV-2 genes detected in aerosols [20]		1–4 μm
Liu et al. (2020): SARS-CoV-2 genes detected in aerosols [21]		<0.25–0.5 μm

## References

[1] Lee B.U. (2020). Minimum sizes of respiratory particles carrying SARS-CoV-2 and the possibility of aerosol generation. Int. J. Environ. Res. Public Health.

[2] Lee B.U. (2021). Why Does the SARS-CoV-2 Delta VOC Spread So Rapidly? Universal Conditions for the Rapid Spread of Respiratory Viruses, Minimum Viral Loads for Viral Aerosol Generation, Effects of Vaccination on Viral Aerosol Generation, and Viral Aerosol Clouds. Int. J. Environ. Res. Public Health.

